# Coupled Effects of Wall Vibration and Surface Wettability on Nanoscale Liquid Film Boiling: A Molecular Dynamics Study

**DOI:** 10.3390/nano16150944

**Published:** 2026-07-31

**Authors:** Haowei Hu, Zhenxin Chen, Feilong Zhao, Qin Li, Lin Guo

**Affiliations:** 1 School of Environment and Energy Engineering, Anhui Jianzhu University, Hefei 230601, China; huhaoweihhw@foxmail.com (H.H.); chenzhenxin0519@163.com (Z.C.); 17856770708@163.com (F.Z.); 2 School of Materials and Chemical Engineering, Anhui Jianzhu University, Hefei 230601, China; 3 School of Energy and Power Engineering, Shandong University, Jinan 250061, China

**Keywords:** nanoscale boiling, molecular dynamics simulation, surface vibration, Leidenfrost effect, wettability

## Abstract

Nanoscale liquid film boiling is a key heat transfer mechanism in high-heat-flux thermal management, but the coupled effects of wall vibration and surface wettability remain unclear. In this study, molecular dynamics simulations were performed to investigate water film boiling on platinum surfaces under different wettability conditions, vibration amplitudes, and vibration frequencies. The results show that surface wettability strongly regulates the balance between early nucleation and later heat transfer deterioration. Under wall vibration, the neutral-wettability case (*β* = 0.02) shows the most favorable overall behavior, with bubble nucleation occurring at 0.35 ns, 46.2% earlier than that for *β* = 0.013, while the Leidenfrost onset is delayed to 1.65 ns, 153.8% later than that for *β* = 0.1. For vibration amplitude, increasing the amplitude from 0.5 to 2 Å advances bubble nucleation by 70.8%, whereas further increasing the amplitude to 3–4 Å accelerates Leidenfrost onset by 51.5–75.8%. For vibration frequency, compared with the non-vibrating case, nucleation is advanced by 36.4%, 54.5%, and 81.8% at 100, 150, and 200 GHz, respectively. These results indicate that moderate vibration enhances interfacial energy exchange, whereas excessive vibration promotes premature vapor-layer formation and heat transfer deterioration.

## 1. Introduction

Against the background of carbon peaking and carbon neutrality, improving the energy conversion and utilization efficiency of various devices has become a primary task for sustainable development. Most of these devices rely on working fluids to transfer and convert thermal energy, such as power generation systems, nuclear reactors, air-conditioning systems, water harvesting devices, high-power electronic devices, and battery thermal management systems [1,2,3]. Boiling heat transfer has become an efficient heat transfer mode because a large amount of latent heat of vaporization is released during the phase-change process [4,5], enabling a high heat transfer coefficient at a relatively low wall superheat. With the rapid development of micro- and nanoelectromechanical systems and high-integration chips, the characteristic dimensions of devices have already entered the nanoscale, resulting in a sharp increase in heat flux [6,7]. Conventional single-phase heat transfer methods can hardly meet the demand under high-heat-flux conditions. As a key breakthrough for next-generation high-efficiency cooling technologies, nanoscale boiling phase-change heat transfer urgently requires clarification of its microscopic physical mechanisms.

Boiling heat transfer enhancement technologies can be classified into active and passive enhancement techniques. Active enhancement techniques are mainly based on externally applied energy fields, such as gas injection [8], magnetic fields [9], electric fields [10], and ultrasonic vibration [11]. Passive enhancement techniques focus on modifying the heat transfer surface or the thermophysical properties of the working fluid, including the construction of nanostructured surfaces, such as nanorough surfaces, nanoporous surfaces, and nanomaterial coatings, the use of nanofluids, and surface functionalization or wettability regulation [12,13,14,15]. To analyze phase-change heat transfer at the micro- and nanoscale and to reveal the microscopic mechanisms of boiling heat transfer in active and passive enhancement techniques, molecular dynamics simulation has become one of the effective methods for micro- and nanoscale investigations. To date, numerous review articles on nucleate boiling have been published. These reviews mainly focus on the following topics: (1) theoretical and empirical models of boiling heat transfer [16,17]; (2) various boiling heat transfer enhancement techniques, such as nanofluids, surface modification, external fields, and porous structures [18,19,20]; (3) research progress on bubble dynamics and nucleation mechanisms [21,22]; and (4) reviews of numerical studies across different scales [23,24,25]. Molecular dynamics simulation can describe the motion characteristics of fluids with atomic-level precision, determine the trajectory of each atom, and effectively obtain heat transfer characteristics at micro- and nanoscale dimensions. Therefore, it has become an important tool for investigating boiling phenomena in nanoscale systems.

Shavik et al. [26] investigated the boiling behavior of liquid argon nanofilms under different wetting conditions using molecular dynamics simulations. Their results showed that increasing wall lyophilicity can reduce the energy barrier for bubble nucleation, thereby promoting bubble nucleation, whereas lyophobic walls exhibit weaker interactions with liquid molecules, making discontinuous separation more likely to occur at the solid–liquid interface. Cao et al. [27] constructed a composite wall model with lyophilic-lyophobic patterned regions using molecular dynamics simulations. They found that during liquid film boiling under heating, increasing the proportion of lyophilic regions on the heating wall reduced the solid–liquid interfacial thermal resistance, strengthened interfacial interactions, and significantly improved heat transfer efficiency. Zhang et al. [28] compared boiling behavior on different micro- and nanostructured walls through simulations. Their results indicated that both concave and convex structures can significantly promote bubble nucleation and advance the bubble nucleation time, while concave structures exhibit a faster development rate during the nucleate boiling stage. Zhou et al. [29] found that rectangular cavity structures can significantly advance the bubble nucleation time, and cavities with smaller widths exhibit stronger boiling heat transfer enhancement because they are more likely to induce local heat accumulation.

Hu et al. [30] proposed a pressure control method based on molecular dynamics simulations, providing insights into the mechanisms by which system pressure affects bubble formation, interfacial evolution, and the nucleation energy barrier during nucleate boiling under complex pressure environments, thereby offering a reference for the study of boiling heat transfer mechanisms. Li Yun et al. [31] modified the wettability of condensation surfaces, which altered the interactions between the membrane surface and water molecules as well as the phase-change process and discharge behavior of condensate in nanopores. Their study revealed the influence of membrane surface wettability on the condensation process, migration behavior, and transport efficiency of water molecules at the molecular scale. Hu Haowei et al. [32] investigated water vapor condensation and infiltration in nanopores with tunable wettability and revealed the regulatory effect of wall wettability gradients on water molecule adsorption, condensation, and transport efficiency. Deng et al. [33] and Wang et al. [34,35] further demonstrated, from the perspectives of surface temperature, film thickness, and wettability, that lyophilic/lyophobic surfaces have coupled effects on interfacial thermal resistance, temperature gradients, and the onset time of explosive boiling. Ilic et al. [36] and Mao et al. [37] further revealed the staged evolution characteristics of near-wall superheating, bubble coalescence, vapor-film lift-off, and heat transfer deterioration during rapid boiling of water films. Meanwhile, the effects of nanowalls, nanopillars, and nanostructured surfaces on the triggering of explosive boiling and evaporation rates have also been verified through molecular dynamics simulations [38,39].

Although numerous molecular dynamics studies have been conducted on nanoscale liquid film boiling, research on vibration-regulated boiling heat transfer remains relatively limited. Pillai et al. [40] investigated the acoustothermal atomization of water nanofilms using molecular dynamics simulations and demonstrated that high-frequency, sub-nanometer surface vibration can induce strong interfacial perturbations and promote liquid–vapor phase transition at the nanoscale. Their work indicates that under nanoscale confinement, vibration-induced energy transfer and viscous dissipation can significantly affect the stability and breakup of liquid nanofilms. In addition, quasi-two-dimensional molecular dynamics models have been used as an efficient strategy for nanoscale boiling simulations. Liu et al. [41] adopted a simulation domain of approximately 122.9 Å × 21.7 Å × 657.9 Å, where the narrow transverse dimension is comparable to the 20 Å used in the present study. Such a configuration can reduce computational cost while retaining the main liquid–vapor interfacial evolution in the heat transfer plane.

Based on the analysis of previous studies, both active and passive heat transfer enhancement techniques have important effects on the boiling process of nanoscale liquid films. However, studies on boiling heat transfer characteristics under vibration remain relatively insufficient, and the boiling process of nanoscale liquid films under the coupled effects of dynamic wall vibration and wettability has not yet been investigated. In this study, a vibration–wettability coupled regulation model was established using molecular dynamics simulations to further reveal the regulatory mechanisms of the synergistic effects of vibration and wettability on bubble nucleation, interfacial evolution, and the transition to explosive boiling. Building upon existing studies on liquid film boiling involving vibration, this work investigates the boiling heat transfer behavior of nanoscale water films under different vibration amplitudes and frequencies, and discusses the intrinsic mechanisms of vibration-induced heat transfer enhancement and heat transfer deterioration at the microscopic scale.

## 2. Materials and Methods

Molecular dynamics simulations of nanoscale water film boiling on platinum substrates were performed using the Large-scale Atomic/Molecular Massively Parallel Simulator (LAMMPS, version 2 Aug 2023, Sandia National Laboratories, Albuquerque, NM, USA) [42], and the simulation trajectories were visualized and analyzed using OVITO Pro (version 3.10.6, OVITO GmbH, Darmstadt, Germany) [43].

The simulation box dimensions are 20 nm (*x*) × 2 nm (*y*) × 52.2 nm (*z*). Periodic boundary conditions are applied in the *x*- and *y*-directions to represent an infinitely extended planar liquid film. Since the length in the *y*-direction is much smaller than those in the *x*- and *z*-directions, the system can be regarded as a quasi-two-dimensional model. This configuration captures the main physical processes during liquid film boiling, including bubble nucleation, growth, coalescence, and vapor-film formation, while reducing the computational cost of molecular dynamics simulations.

**Figure 1:**
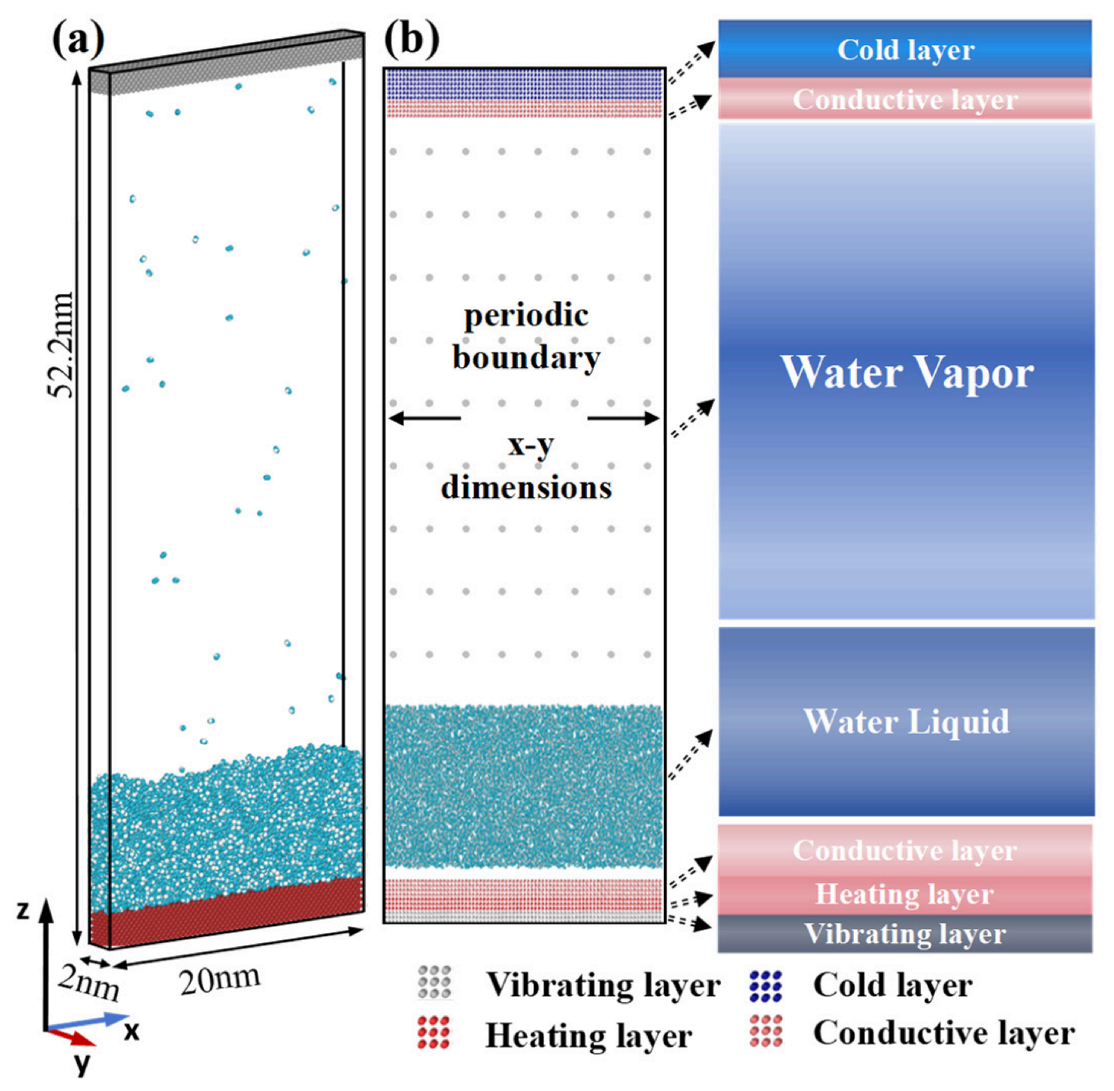
Schematic of the simulated system: (**a**) three-dimensional atomic configuration, system dimensions, and coordinate system; (**b**) arrangement of the functional layers and boundary conditions, including the bottom vibration, heating, and heat-conduction layers, the liquid-water and water-vapor regions, and the top heat-conduction and cold layers. Periodic boundary conditions are applied in the *x* and *y* directions, while the *z* direction is normal to the solid–liquid interface.

The model consists of seven functional regions from bottom to top: the bottom vibration layer with a height of 5.88 Å, the bottom heating layer with a height of 5.88 Å, the bottom Pt heat-conduction layer with a height of 7.84 Å, the liquid water region with a height of 70 Å, the water vapor diffusion region, the top Pt heat-conduction layer with a height of 7.84 Å, and the top cold layer with a height of 13.72 Å, as shown in Figure 1a,b. The bottom vibration layer is used to apply external vibration excitation to the solid substrate. The bottom heating layer is maintained at 700 K to supply heat to the system, while the bottom heat-conduction layer transfers heat from the heating layer to the liquid film region. The liquid water region serves as the boiling working fluid, and the water vapor diffusion region provides space for liquid film evaporation and bubble growth. The top cold layer is maintained at 300 K to provide a low-temperature boundary at the top of the system.

A 7 nm liquid water layer, consisting of 25,854 rigid TIP4P/2005 water molecules, is maintained in the simulation system. The solid Pt substrate is arranged in a face-centered cubic (FCC) lattice with a lattice constant of 3.92 Å. A vibration layer is placed at the bottom of the substrate. Surface vibration excitation in the z-direction is achieved by imposing a prescribed sinusoidal motion on the bottom three layers of Pt atoms. The vibration is first applied to the bottom Pt atoms and then transmitted to the entire solid substrate through interlayer interactions among Pt atoms, thereby inducing dynamic disturbances in the liquid water film above.

The embedded-atom method (EAM) was adopted to accurately describe the interatomic interactions among Pt atoms. The intramolecular constraints of water molecules were treated using the TIP4P/2005 model, while the water–water and water–Pt interactions were described by the Lennard-Jones (LJ) potential and its modified form, respectively [44]:

(1) $$\Phi (r)=4\varepsilon [{\left(\frac{\sigma }{r}\right)}^{12}-{\left(\frac{\sigma }{r}\right)}^{6}]$$

To precisely regulate the wettability of the solid–liquid interface, a modified Lennard-Jones (LJ) potential was adopted in this study to describe the water–Pt interactions:

(2) $$\Phi (r)=4\varepsilon_{sl}[{\left(\frac{\sigma_{sl}}{r}\right)}^{12}-\beta {\left(\frac{\sigma_{sl}}{r}\right)}^{6}]$$

(3) $$\varepsilon_{sl}=\alpha \sqrt{\varepsilon_{s}\varepsilon_{l}}$$

(4) $$\sigma_{sl}=\frac{\sigma_{s}+\sigma_{l}}{2}$$

where *ε* and *σ* are the energy and length parameters, respectively, and *r* is the interatomic distance. The subscripts s and l denote the solid and liquid phases, respectively. The Lennard-Jones energy parameter *ε* was used to describe the interaction strength between water molecules and Pt atoms. In this work, the wettability of the bottom Pt wall was adjusted by varying the O-Pt energy parameter, denoted as *β* for convenience. A larger *β* indicates stronger solid–liquid interactions and a more hydrophilic surface. Following established molecular dynamics approaches, the corresponding macroscopic wetting states were determined through equilibrium droplet contact-angle simulations rather than directly assigned from *β* [35,45]. Within the investigated parameter range, a larger *β* strengthens the solid–liquid interaction, decreases the equilibrium contact angle, and consequently results in a more hydrophilic surface.

**Figure 2:**
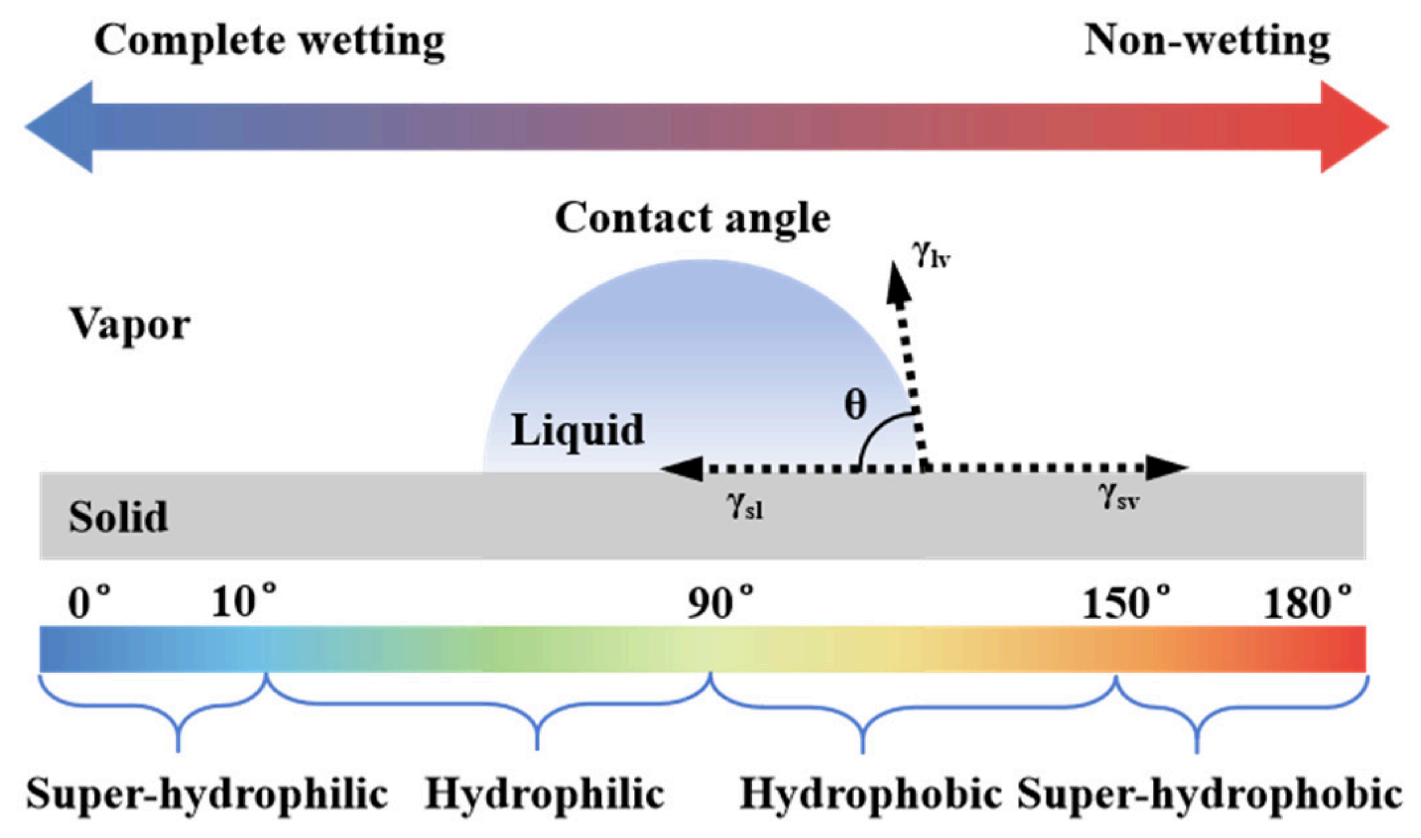
Schematic definition of the equilibrium contact angle used to characterize surface wettability.

As shown in Figure 2, the selected potential parameters used for the water–water, water–Pt, and Pt–Pt interactions in the molecular dynamics simulations are summarized in Table 1. Pt_down_ and Pt_up_ denote the bottom Pt wall and the top Pt wall, respectively.

**Figure 3:**
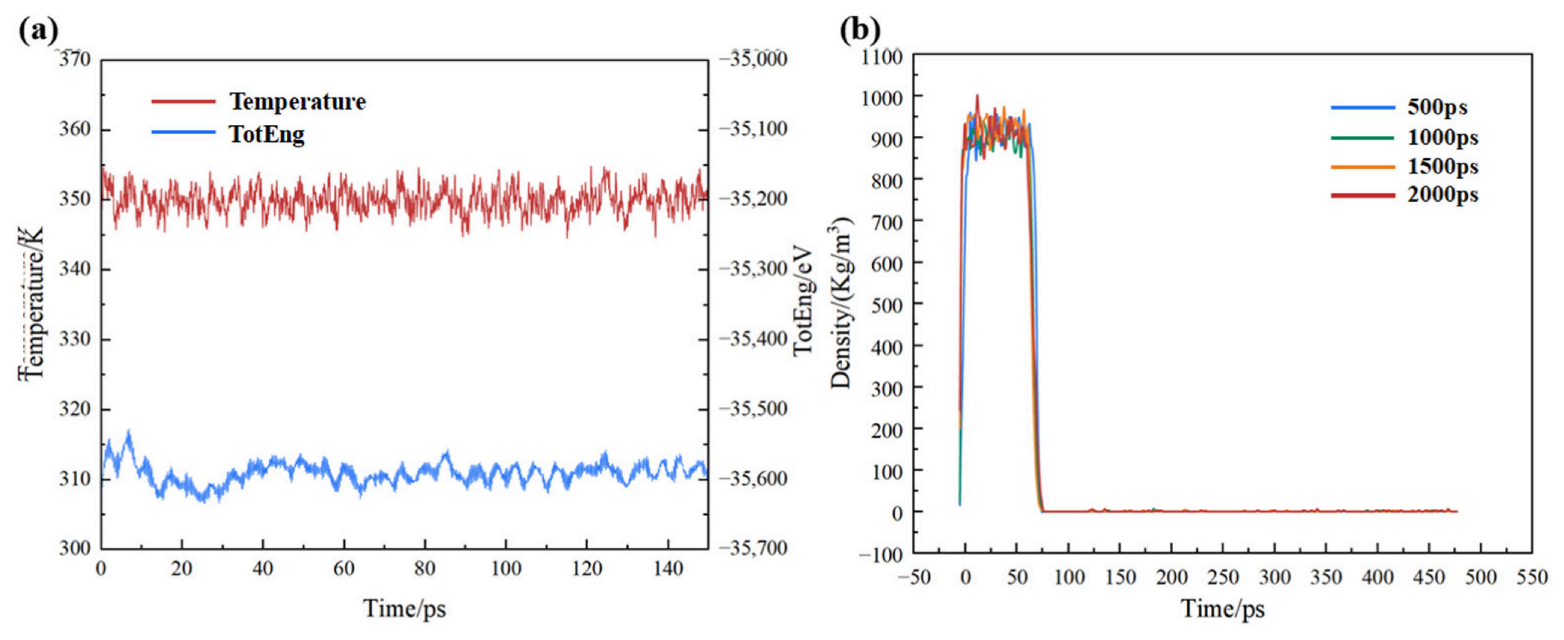
(**a**) Variations in liquid film temperature and energy with time during the equilibrium stage and (**b**) variations in density distribution of the liquid film along the *z*-axis.

The simulation was performed in two stages: relaxation and heating. During the relaxation stage, the entire system was equilibrated in the NVT ensemble at 370 K for 1.5 ns with a time step of 5 fs. The system was considered to have reached thermodynamic equilibrium when the liquid film temperature, total energy, and density distribution became stable, as shown in Figure 3.

**Figure 4:**
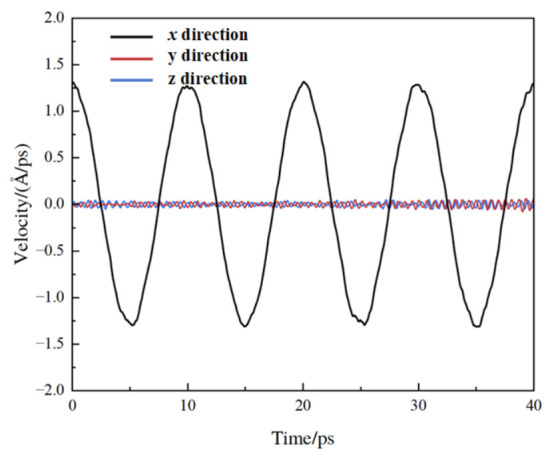
Time evolution of the three-dimensional velocity components of the substrate during the vibration verification simulation.

During the heating stage, the bottom heating layer was maintained at 700 K as a constant-temperature heat source to simulate heating under high-heat-flux conditions. Meanwhile, vibration in the *z*-direction was applied to the Pt atoms in the bottom vibration layer, and the corresponding displacement equation is given as follows:

(5) $$\omega =2\pi f=\frac{2\pi }{T}$$

(6) $$z\left(t\right)=z\left(t_{0}\right)+A_{Z}sin\left(\omega t\right)$$

where $z(t_{0})$ is the initial atomic position, A is the amplitude vector, ω is the angular frequency, f is the vibration frequency, and T is the vibration period. Since the external vibration is directly imposed only on the bottom three Pt layers, while it can propagate to other atoms in the substrate through interatomic interactions, it is necessary to examine whether the prescribed simple harmonic motion of these atoms can excite vibration throughout the entire substrate. In this study, the vibration amplitude and frequency of the Pt atoms in the bottom three layers were set to 2 Å and 100 GHz, respectively, where 100 GHz is equivalent to $1\times 10^{11}$ Hz. To verify whether the imposed vibration can be transmitted through the solid substrate, an independent substrate vibration test was performed before the boiling simulations. In this test, the substrate temperature was maintained at 370 K. The velocities of Pt atoms in the substrate were sampled during this vibration-only stage. As shown in Figure 4, although the external vibration was directly imposed on the bottom vibration layer, a periodic velocity response was observed throughout the substrate, indicating that the vibration can be effectively transferred through the solid Pt wall.

## 3. Results and Discussion

### 3.1. Mechanism Analysis of Vibration Effects on the Enhancement-Deterioration of Boiling Heat Transfer

First, a model with an amplitude of *A* = 2 Å, a wall temperature of 700 K, and a vibration frequency of *f* = 100 GHz was selected for the applicability test. The wall wettability was set to a relatively hydrophilic condition (*β* = 0.02, *θ* = 68°), under which the influence of vibration on the liquid film boiling process can be more readily observed.

**Figure 5:**
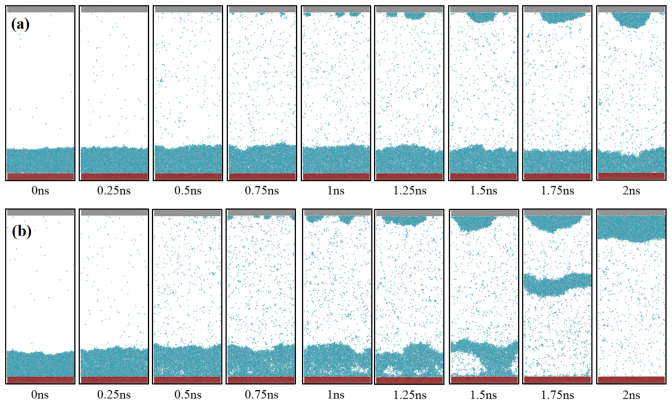
(**a**) Simulation snapshots under the non-vibration condition; (**b**) simulation snapshots under the vibration condition. White and cyan particles represent the oxygen and hydrogen atoms of water molecules, respectively, while red and dark-gray particles represent Pt atoms in the lower heated substrate and upper cooled wall, respectively.

The simulation processes under vibrating and non-vibrating conditions are shown in Figure 5. Under the non-vibrating condition, bubble generation, departure, and coalescence occur relatively slowly. The interface remains comparatively stable, and no continuous vapor film is formed within the simulated time window. In contrast, under the vibrating condition, bubble nucleation becomes more frequent, and bubble departure, coalescence, and breakup occur more readily, indicating pronounced interfacial instability and an increased renewal rate of the gas–liquid interface. After the introduction of wall vibration, the bubbles in the liquid film boiling process coalesce near the wall at approximately 1.65 ns, forming a relatively stable vapor layer, namely, the occurrence of the Leidenfrost effect. This phenomenon is consistent with the findings reported by He et al. [46].

To clarify the physical meaning and calculation method of the instantaneous heat current, the microscopic heat current formulation was used to characterize transient energy transport along the heat transfer direction. The heat current vector can be expressed as:

(7) $$J=\sum_{i}e_{i}v_{i}-\sum_{i}S_{i}V_{i}$$

(8) $$e_{i}=KE_{i}+PE_{i}$$

where *e_i_* is the total energy of atom *i*, including kinetic energy *KE_i_* and potential energy *PE_i_*; *v_i_* is the atomic velocity; and *S_i_* is the per-atom stress tensor. In this work, the *z*-direction component of the heat current is used to characterize the transient thermal response. The instantaneous heat current *J* is calculated for the water phase based on the microscopic heat current expression. It represents the net transient energy-transport response of the whole confined water film, rather than a lower-wall-resolved interfacial heat transfer quantity. Because the water film is confined between the lower heated wall and the upper cold wall, *J* may reflect the combined effects of lower-wall heating, upper-wall cooling, liquid–vapor phase transition, and transient energy redistribution within the water phase. *J* is not normalized by the heat transfer area and is therefore reported as an instantaneous heat current in eV·Å/ps, rather than as an area-normalized quantity.

**Figure 6:**
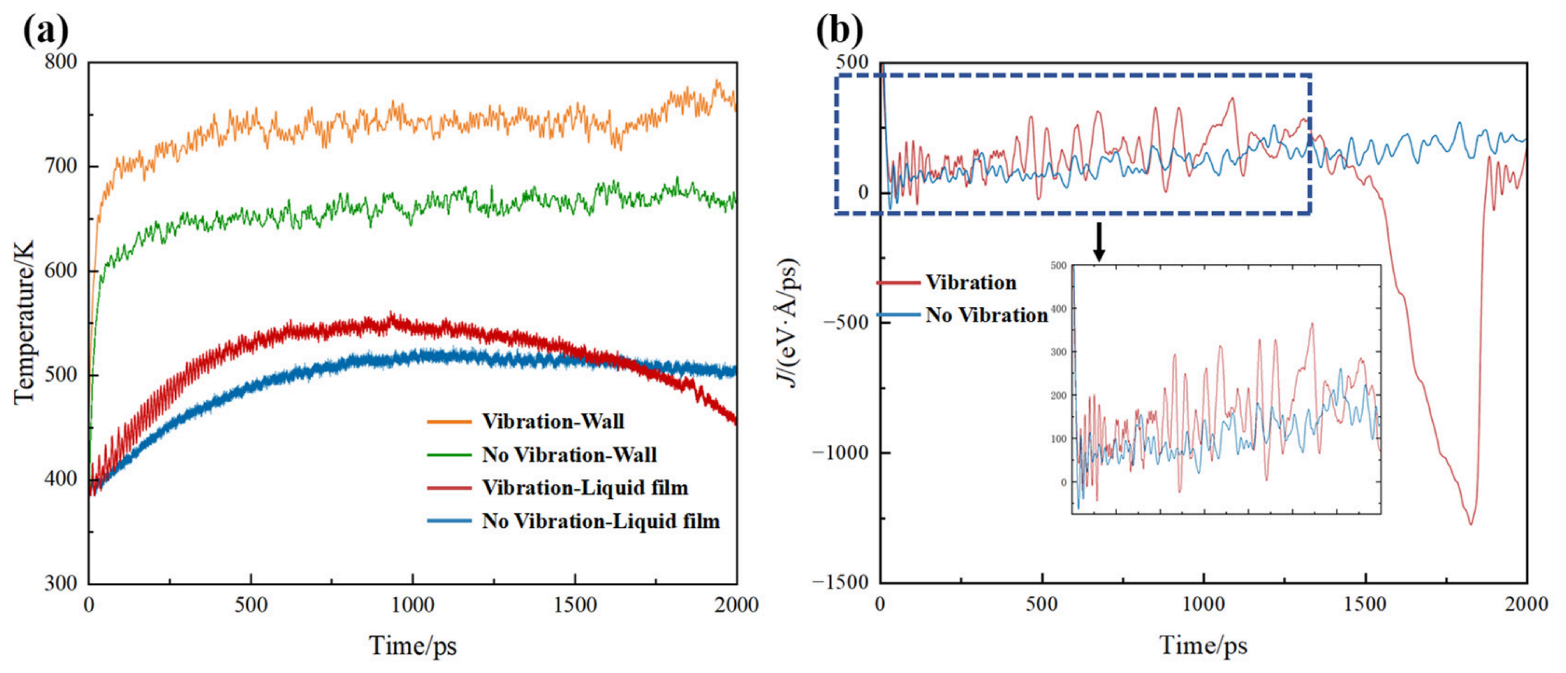
Variations in (**a**) the temperatures of the substrate and liquid film with time and (**b**) the instantaneous heat current with time under vibration and non-vibration conditions. Vibration accelerates the early temperature response by enhancing interfacial disturbance and energy transport, but the resulting rapid bubble coalescence promotes continuous vapor-layer formation and subsequent heat transfer deterioration.

Figure 6 shows the temporal evolution of the regional temperature and instantaneous heat current *J* under vibrating and non-vibrating conditions. From 0 to 1.45 ns, *J* is generally higher under wall vibration, indicating that vibration enhances the transient energy-transport response of the confined liquid film. After approximately 1500 ps, *J* first decreases gradually and then drops sharply to large negative values. The gradual decrease is associated with the increased effective thermal resistance caused by the formation of a Leidenfrost-like vapor layer. The sharp negative response reflects transient energy redistribution during vapor-layer formation, liquid film detachment, liquid–vapor phase transition, and interaction with the upper cold boundary.

The bubble dynamics analysis indicates that wall vibration enhances early-stage heat transfer by increasing bubble nucleation density and coalescence rate, thereby accelerating vapor-phase development and the formation of a continuous vapor layer. In the non-vibrating case, the temperature increases more steadily, and a stable insulating layer is less readily formed within the same time window, resulting in milder variations in temperature and *J*. Thus, vibration enhances early interfacial energy transport while also promoting vapor-layer formation at later stages, which eventually weakens heat transfer.

### 3.2. Coupled Effects of Surface Wettability and Vibration on Boiling Heat Transfer Characteristics

#### 3.2.1. Effect of Surface Wettability

**Figure 7:**
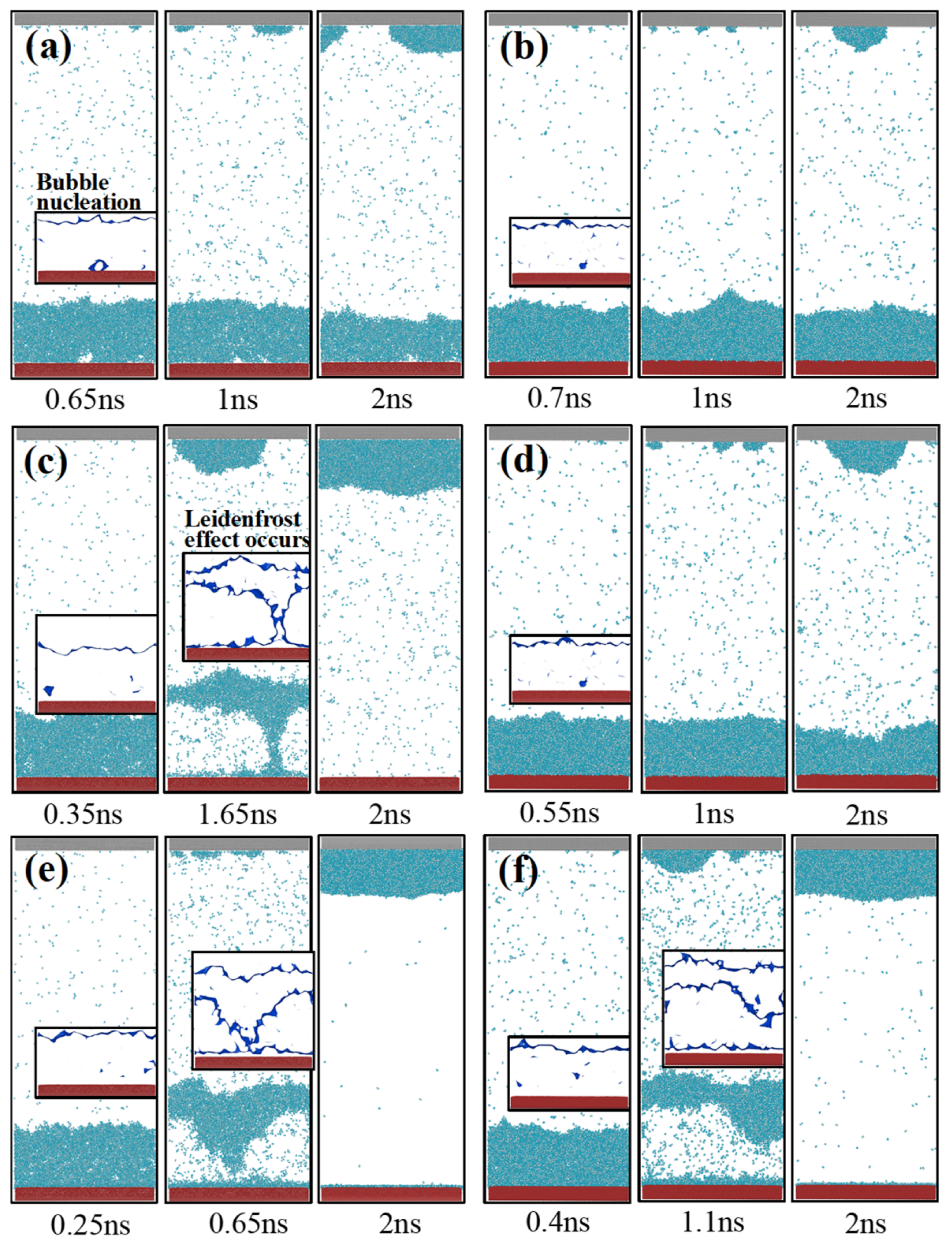
Liquid film boiling snapshots under different wettability conditions with and without wall vibration. Panels (**a**,**c**,**e**) show vibration cases, and panels (**b**,**d**,**f**) show non-vibration cases. Panels (**a**,**b**), (**c**,**d**), and (**e**,**f**) correspond to *β* = 0.013, 0.02, and 0.1, respectively. White and cyan particles represent the oxygen and hydrogen atoms of water molecules, respectively, while red and dark-gray particles represent Pt atoms in the lower heated substrate and upper cooled wall, respectively. The insets indicate bubble nucleation and, where present, Leidenfrost onset; the irregular blue contour in each inset denotes the liquid–vapor boundary.

Figure 7 shows the evolution of the liquid film boiling process under three wettability conditions with and without wall vibration.

**Figure 8:**
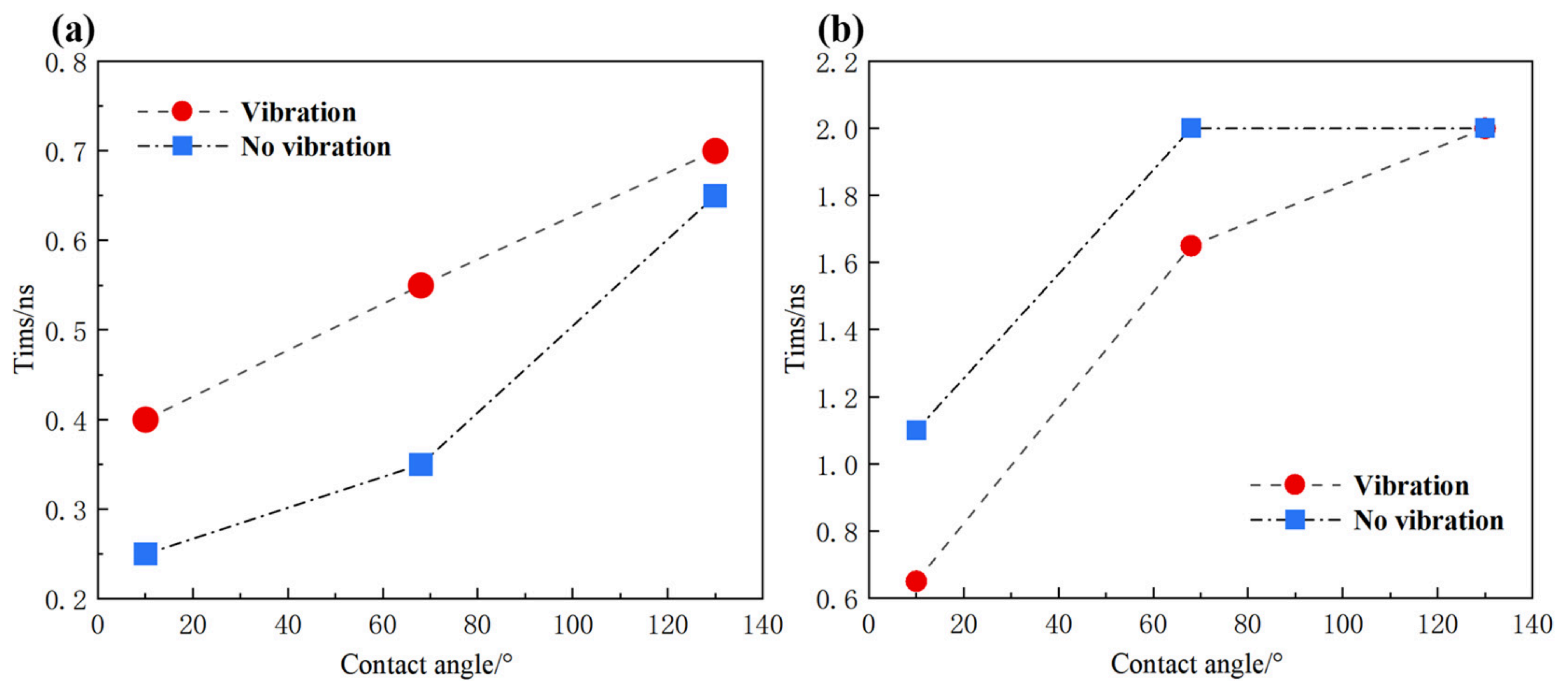
(**a**) Bubble nucleation onset under different wettability conditions and (**b**) onset time of the Leidenfrost effect under different wettability conditions.

In Figure 7, panels (a, c, e) correspond to the vibration cases, whereas panels (b, d, f) correspond to the non-vibration cases; panels (a, b), (c, d), and (e, f) represent *β* = 0.013, 0.02, and 0.1, respectively. Here, *β* = 0.013 corresponds to a hydrophobic surface, *β* = 0.02 corresponds to a hydrophilic surface, and *β* = 0.1 corresponds to a super hydrophilic surface. The insets mark the characteristic boiling events, with the first inset indicating bubble nucleation, and the second inset, when present, indicating the onset of the Leidenfrost effect. The moments of bubble nucleation and explosive boiling were identified using surface-mesh-based post-processing, as shown in Figure 8. It should be noted that the characteristic times of bubble nucleation and Leidenfrost onset reported in this study are extracted from reproducible deterministic trajectories under fixed initial configurations. Therefore, these times are used mainly as comparative indicators among different simulation conditions, rather than ensemble-averaged quantities with statistical uncertainty. By analyzing the variations in the onset times of bubble nucleation and explosive boiling during the boiling process, it can be found that, owing to the vibration-induced perturbation, the variation in bubble nucleation time under different wettability conditions is relatively small. In contrast, under the non-vibrating condition, the nucleation time is more strongly affected by surface wettability. An opposite trend is observed for the explosive boiling time: the fluctuation in the onset time under the vibrating condition is much larger than that under the non-vibrating condition, with a maximum difference of 0.45 ns. Moreover, regardless of whether wall vibration is applied, the bubble nucleation time advances significantly with increasing surface wettability, indicating that enhanced hydrophilicity promotes bubble nucleation. This result is consistent with the findings reported by Gao [47].

**Figure 9:**
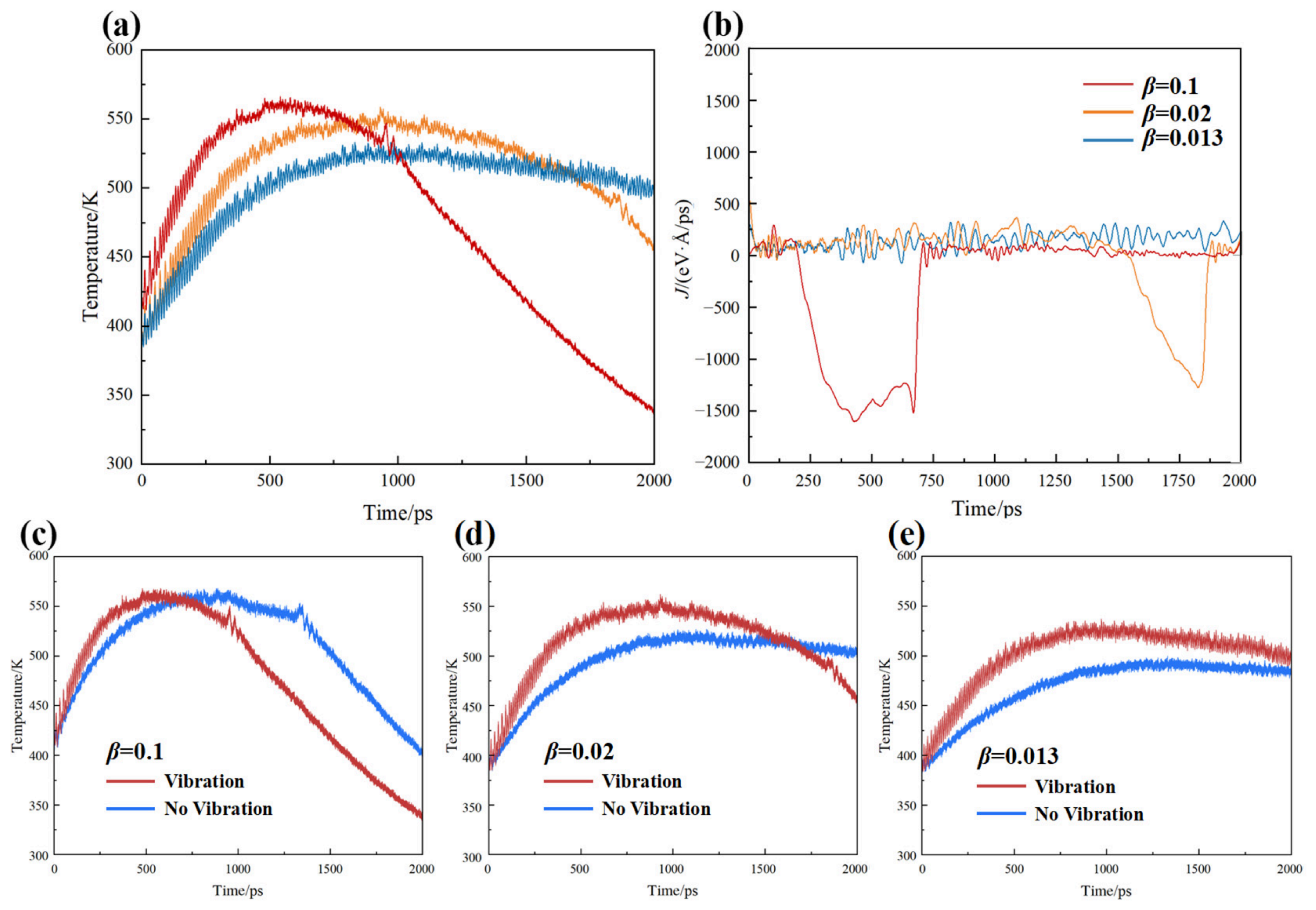
Effects of surface wettability and wall vibration on the thermal response of the liquid film. (**a**) Time evolution of the liquid film temperature under wall vibration with different wettability parameters. (**b**) Time evolution of the instantaneous heat current under wall vibration with different wettability parameters. (**c**–**e**) Comparison of liquid film temperature with and without wall vibration under different wettability conditions: (**c**) *β* = 0.1, (**d**) *β* = 0.02, and (**e**) *β* = 0.013. Increasing *β* strengthens the water–Pt interaction and therefore accelerates the initial temperature rise and bubble nucleation. At larger *β*, the earlier formation of a continuous vapor layer interrupts direct contact with the heated wall, resulting in an earlier temperature decrease and heat transfer deterioration.

Figure 9 further compares the liquid film temperature and instantaneous heat current responses under different wettability conditions. Quantitatively, under wall vibration, the bubble nucleation time decreases from 0.65 ns at *β* = 0.013 to 0.35 ns at *β* = 0.02 and 0.25 ns at *β* = 0.1, corresponding to advances of 46.2% and 61.5%, respectively. Without vibration, as *β* increases from 0.013 to 0.02 and 0.1, the nucleation time decreases from 0.70 ns to 0.55 ns and 0.40 ns, corresponding to advances of 21.4% and 42.9%, respectively. These results confirm that stronger solid–liquid interactions promote bubble nucleation with or without wall vibration. Stronger wettability also accelerates vapor-layer formation and heat transfer deterioration. Under lyophobic conditions, explosive boiling does not occur within the simulated time window, and wall vibration slightly improves the liquid film temperature response. Under lyophilic conditions, explosive boiling occurs in both vibrating and non-vibrating cases, while the vibrating case enters the Leidenfrost state earlier. After the liquid film detaches from the heated wall and interacts with the upper cold layer, the liquid film temperature decreases rapidly, accompanied by a pronounced negative response in *J*. Under intermediate-wettability conditions, the system is more sensitive to vibration: the Leidenfrost onset occurs at approximately 1.65 ns under vibration, whereas no continuous vapor film forms within 2 ns without vibration. Therefore, *β* = 0.02 provides a better balance between promoting early bubble nucleation and delaying premature Leidenfrost transition.

As the surface changes from hydrophobic to hydrophilic, the interfacial heat transfer capability is enhanced, causing bubble nucleation and explosive boiling to occur earlier overall. Under the coupled effects of wettability and vibration at the nanoscale, interfacial perturbations can be intensified, thereby modulating bubble coalescence and vapor-layer formation. Among the three wettability conditions, applying vibration under neutral wettability shows a significant effect on optimizing boiling heat transfer.

#### 3.2.2. Effect of Vibration Amplitude

**Figure 10:**
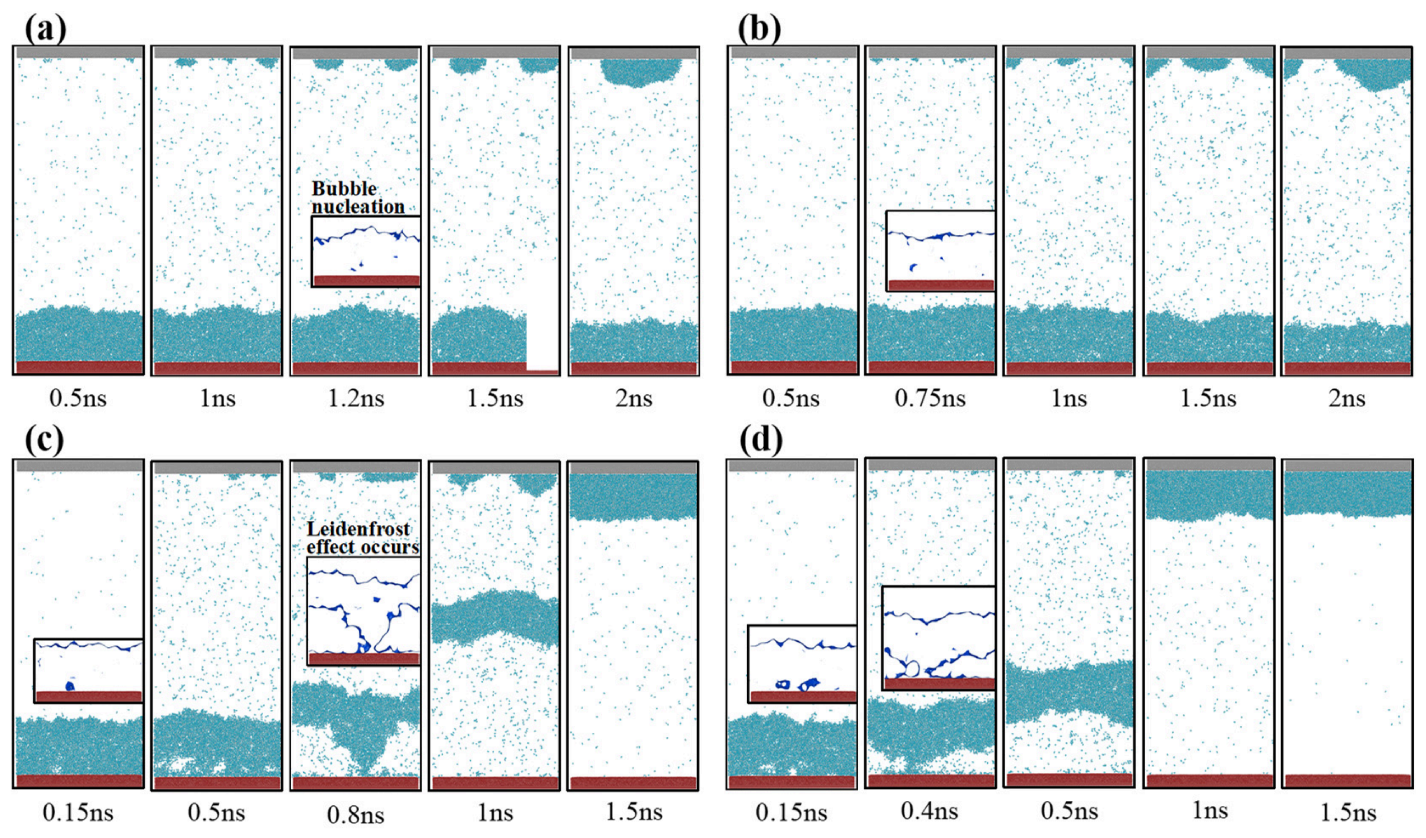
Simulation snapshots of nanoscale water film boiling under different vibration amplitudes, with the onset times of bubble nucleation and explosive boiling marked: (**a**) *A* = 0.5 Å; (**b**) *A* = 1 Å; (**c**) *A* = 3 Å; and (**d**) *A* = 4 Å. White and cyan particles represent the oxygen and hydrogen atoms of water molecules, respectively, while red and dark-gray particles represent Pt atoms in the lower heated substrate and upper cooled wall, respectively.

To investigate the effect of vibration amplitude on liquid film boiling under wall vibration, five simulation cases were comparatively analyzed, with vibration amplitudes A of 0.5 Å, 1 Å, 2 Å, 3 Å, and 4 Å, while the wettability parameter β and vibration frequency f were kept unchanged. Figure 10 presents the simulation snapshots under different vibration amplitudes. Since the snapshots for the case with A=2 Å are identical to those shown in Figure 5b, they are not repeated here.

As shown in Figure 10, increasing the vibration amplitude markedly advances bubble nucleation and accelerates vapor-layer formation. Quantitatively, as *A* increases from 0.5 Å to 1, 2, 3, and 4 Å, the bubble nucleation time decreases from 1.20 ns to 0.75, 0.35, 0.15, and 0.15 ns, respectively. Compared with *A* = 0.5 Å, the nucleation time is advanced by 37.5%, 70.8%, and 87.5% for *A* = 1, 2, and 3–4 Å, respectively. At small amplitudes (*A* = 0.5 and 1 Å), the vibration-induced disturbance is weak, and no continuous vapor layer is observed within 2 ns. When *A* increases to 2, 3, and 4 Å, the Leidenfrost onset occurs at 1.65, 0.80, and 0.40 ns, respectively, indicating that larger amplitudes strongly disturb the liquid film and promote an earlier transition to explosive boiling.

**Figure 11:**
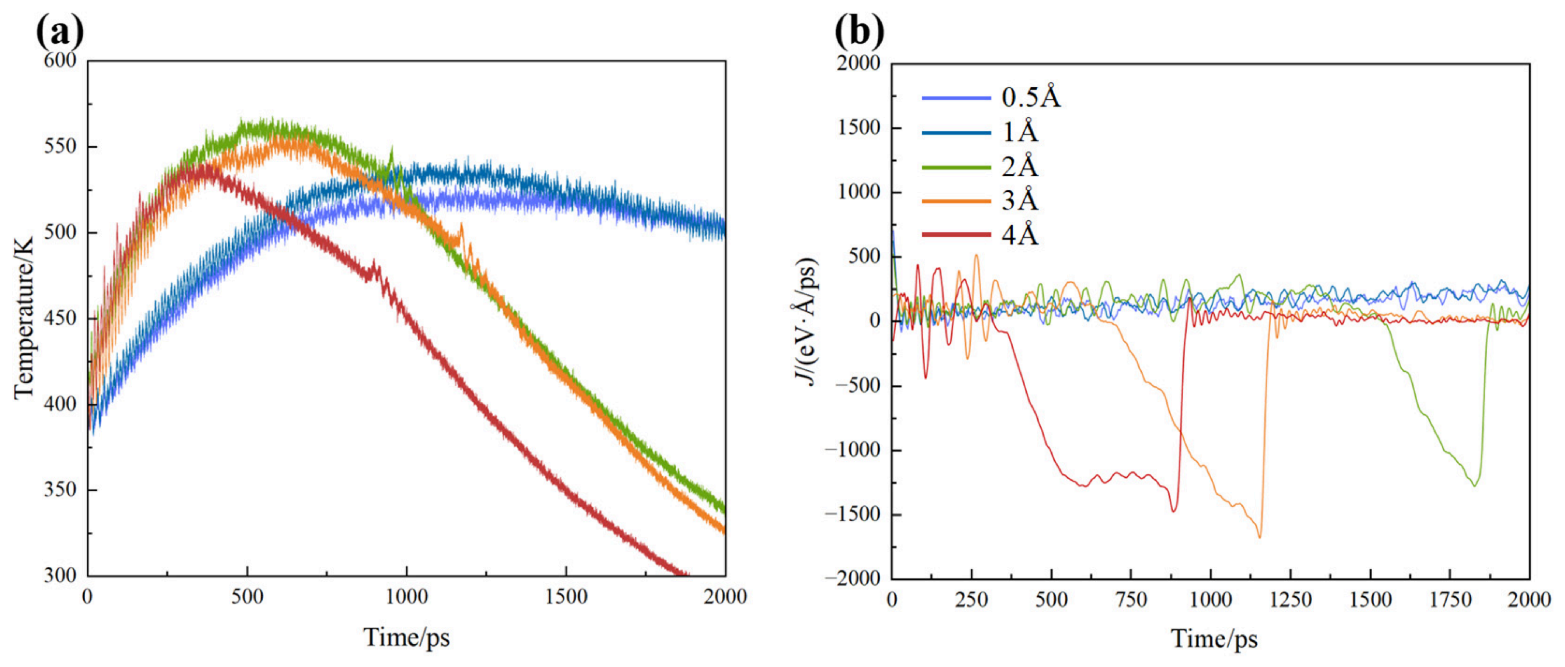
Effects of vibration amplitude on liquid film temperature and heat current. (**a**) Time evolution of the liquid film temperature under different vibration amplitudes. (**b**) Time evolution of the instantaneous heat current under different vibration amplitudes.

As shown in Figure 11, the temperature response further confirms the amplitude-dependent enhancement and deterioration process. The peak liquid film temperature increases from 531.6 K at *A* = 0.5 Å to 545.8 K at *A* = 1 Å and reaches a maximum of 562.9 K at *A* = 2 Å, while the corresponding peak time decreases from 1.223 ns to 1.155 and 0.931 ns, respectively. When *A* increases to 3 Å, the peak temperature remains similar at 563.5 K but appears earlier at 0.692 ns. At *A* = 4 Å, the peak temperature decreases to 544.9 K and occurs at only 0.294 ns, indicating premature vapor-layer formation and rapid heat transfer deterioration. These results show that moderate amplitudes enhance early-stage heat transfer by strengthening vibration-induced interfacial perturbations, whereas excessive amplitudes accelerate the Leidenfrost transition and weaken sustained heat transfer.

#### 3.2.3. Effect of Vibration Frequency

**Figure 12:**
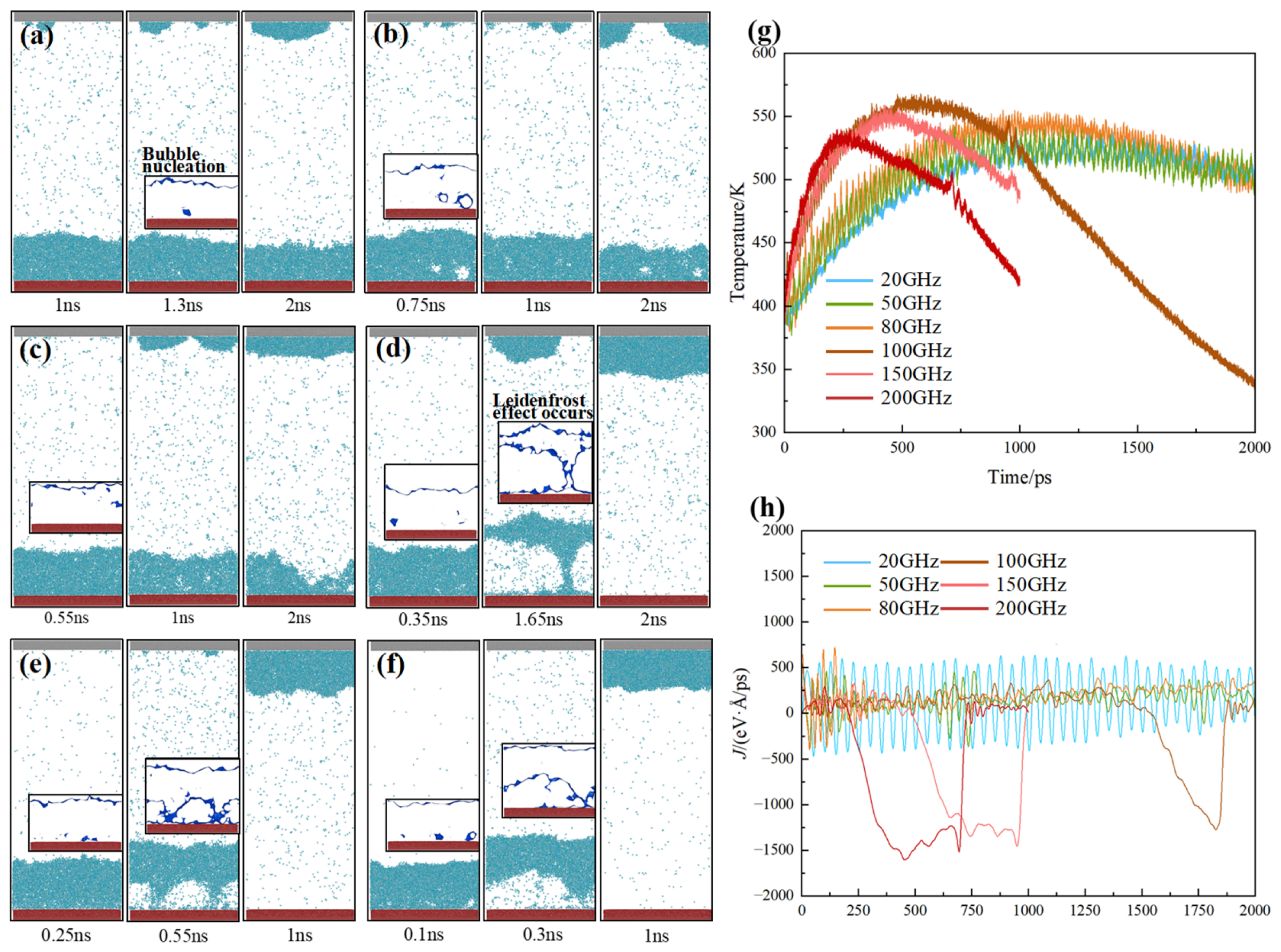
Effects of vibration frequency on water nanofilm boiling and thermal response. (**a**–**f**) Simulation snapshots showing bubble nucleation and Leidenfrost onset under different vibration frequencies: (**a**) 20 GHz, (**b**) 50 GHz, (**c**) 80 GHz, (**d**) 100 GHz, (**e**) 150 GHz, and (**f**) 200 GHz. (**g**) Time evolution of the liquid film temperature. (**h**) Time evolution of the instantaneous heat current under different vibration frequencies. Increasing the vibration frequency intensifies periodic interfacial disturbance and advances bubble nucleation. White and cyan particles represent the oxygen and hydrogen atoms of water molecules, respectively, while red and dark-gray particles represent Pt atoms in the lower heated substrate and upper cooled wall, respectively.

To examine the effect of vibration frequency on liquid film boiling under wall vibration, six vibration frequencies were compared, namely, 20, 50, 80, 100, 150, and 200 GHz, while the wettability parameter and vibration amplitude were kept constant. Figure 12 presents the corresponding simulation snapshots at different vibration frequencies.

As shown in Figure 12a–f, vibration frequency strongly affects the boiling evolution of the liquid film. When the frequency increases from 20 to 50, 80, 100, 150, and 200 GHz, the bubble nucleation time decreases from 1.30 ns to 0.75, 0.55, 0.35, 0.25, and 0.10 ns, respectively. Compared with the non-vibrating case with a nucleation time of 0.55 ns, nucleation is delayed by 136.4% and 36.4% at 20 and 50 GHz, remains nearly unchanged at 80 GHz, and is advanced by 36.4%, 54.5%, and 81.8% at 100, 150, and 200 GHz, respectively. No continuous vapor film is observed within the shown time window at 20–80 GHz, whereas the Leidenfrost onset occurs at 1.65, 0.55, and 0.30 ns at 100, 150, and 200 GHz, respectively.

Figure 12g,h further shows that the peak liquid film temperature first increases with frequency and reaches its maximum at 100 GHz, increasing from 538.5 K at 20 GHz to 562.9 K at 100 GHz, while the peak time generally shifts earlier. At higher frequencies, the peak temperature appears much earlier, at 0.427 ns for 150 GHz and 0.276 ns for 200 GHz, followed by rapid cooling and a pronounced negative net energy-transport response. These results indicate that moderate-frequency vibration enhances early-stage interfacial energy transfer, whereas excessively high frequencies accelerate vapor-layer formation and cause premature heat transfer deterioration.

A comparison of the instantaneous heat current shows that, except for the short initial stage at 200 GHz, approximately from 0 to 0.13 ns, the highest heat current values mainly occur under the low-frequency condition of 20 GHz. The heat current curve at 20 GHz exhibits obvious vibration-induced periodic oscillations, with an oscillation frequency consistent with the imposed vibration. Its overall amplitude shows stable and slight fluctuations, indicating that the system is mainly controlled by vibration-driven reciprocating interfacial perturbations, while bubble generation and development remain limited. In contrast, under higher-frequency conditions, the amplitude of heat current fluctuation increases noticeably in the middle and later stages. This indicates that bubble generation, coalescence, and interfacial reconstruction are markedly intensified, and the heat transfer behavior gradually changes from a vibration-response-dominated process to a strongly unsteady process governed by intense phase change. Therefore, increasing the vibration frequency not only changes the rhythm of interfacial energy input but also promotes the occurrence of explosive boiling in the liquid film.

## 4. Conclusions

In this study, molecular dynamics simulations were performed to systematically elucidate the microscopic physical mechanisms underlying the coupled effects of vibration and surface wettability in nanoscale liquid film boiling. The main conclusions are as follows:

(1) As the heated surface changes from hydrophobic to hydrophilic, bubble nucleation and explosive boiling occur earlier overall. Compared with the effect of wettability variation, the application of vibration has a relatively weaker influence on bubble nucleation. However, wall vibration significantly advances the onset of explosive boiling. Under neutral wettability, vibration can act synergistically with surface wettability to enhance boiling heat transfer.
(2) Wettability, vibration amplitude, and vibration frequency play distinct regulatory roles in nanoscale liquid film boiling. Wettability mainly affects bubble nucleation onset through surface modification, with lyophilic surfaces leading to earlier bubble nucleation and explosive boiling. Vibration amplitude and vibration frequency both enhance interfacial disturbance intensity, thereby accelerating bubble generation and coalescence. As a result, they promote heat transfer at the early stage while also accelerating the onset of explosive boiling. Although low-frequency vibration provides limited heat transfer enhancement compared with conventional heating, its energy input has a distinct periodic character.

## Figures and Tables

**Table 1:** Selected potential parameters.

Interparticle Interaction	Potential Parameters	
	ε (eV)	σ (Å)
Water–Water	0.00802	3.1589
Water–Pt_down_	0.02	2.815
Water–Pt_up_	0.015	2.815
Pt_down_–Pt_down_	EAM	EAM
Pt_up_–Pt_up_	EAM	EAM

## Data Availability

The original contributions presented in this study are included in the article/Supplementary Materials. Further inquiries can be directed to the corresponding author.

## Supplementary Materials

The following supporting information can be downloaded at https://www.mdpi.com/article/10.3390/nano16150944/s1, Figure S1: Initial droplet-on-Pt configuration used for contact-angle calibration; Figure S2: Axisymmetric oxygen-density cloud map and circular interface fitting for *β* = 0.013, corresponding to an apparent contact angle of 130°; Figure S3: Axisymmetric oxygen-density cloud map and circular interface fitting for *β* = 0.02, corresponding to an apparent contact angle of 68°; Figure S4: Axisymmetric oxygen-density cloud map for *β* = 0.1, corresponding to a nearly completely wetting surface with an apparent contact angle of approximately 0°; Table S1: System composition and initial droplet configuration used for contact-angle calibration; Table S2: Pt substrate and water-model parameters; Table S3: O-Pt interaction parameters and calibrated apparent contact angles; Table S4: Parameters used for density-field construction and contact-angle extraction.

## References

[1] Yuan S., Zhao C., Cai X., An L., Shen S., Yan X., Zhang J. (2023). Bubble Evolution and Transport in PEM Water Electrolysis: Mechanism, Impact, and Management. Prog. Energy Combust. Sci..

[2] Fang X., Chen Y., Zhang H., Chen W., Dong A., Wang R. (2016). Heat Transfer and Critical Heat Flux of Nanofluid Boiling: A Comprehensive Review. Renew. Sustain. Energy Rev..

[3] Lin X.W., Zhou Z.F., Yin J., Zhu X.G., Shi M.Y., Chen B. (2024). A Comparative Investigation of Two-Phase Immersion Thermal Management System for Lithium-Ion Battery Pack. J. Clean. Prod..

[4] Lin X.W., Li Y.B., Wu W.T., Zhou Z.F., Chen B. (2024). Advances on Two-Phase Heat Transfer for Lithium-Ion Battery Thermal Management. Renew. Sustain. Energy Rev..

[5] Bai P., Zhou L.P., Du M.H., Wang D., Liu Y., Du X. (2025). Recent Advances in Molecular Dynamics Research on Nanoscale Boiling Heat Transfer. Renew. Sustain. Energy Rev..

[6] Cao Q., Shao W., Cui Z. (2020). Molecular Dynamics Simulations and Mathematical Optimization Method for Surface Structures Regarding Evaporation Heat Transfer Enhancement at the Nanoscale. Int. J. Heat Mass Transf..

[7] Liu H., Deng W., Ding P., Zhao J. (2021). Investigation of the Effects of Surface Wettability and Surface Roughness on Nanoscale Boiling Process Using Molecular Dynamics Simulation. Nucl. Eng. Des..

[8] Gose E.E., Peterson E.E., Acrivos A. (1957). On the Rate of Heat Transfer in Liquids with Gas Injection through the Boundary Layer. J. Appl. Phys..

[9] Zhou L.J. (2010). Multi-Scale Study on Flow and Energy Transfer Characteristics of Magnetic Fluids. Ph.D. Dissertation.

[10] Shahriari A., Birbarah P., Oh J., Miljkovic N., Bahadur V. (2017). Electric Field Based Control and Enhancement of Boiling and Condensation. Nanoscale Microscale Thermophys. Eng..

[11] Bulliard-Sauret O., Ferrouillat S., Vignal L., Memponteil A., Gondrexon N. (2017). Heat Transfer Enhancement Using 2 MHz Ultrasound. Ultrason. Sonochem..

[12] Zhou W., Li Y., Li M., Wei J., Tao W. (2019). Bubble Nucleation over Patterned Surfaces with Different Wettabilities: Molecular Dynamics Investigation. Int. J. Heat Mass Transf..

[13] Liu Z., Liu Z., Liu R. (2023). Rapid Boiling of Ultra-Thin Liquid Argon Film on Patterned Wettability Surface with Nanostructure: A Molecular Dynamics Investigation. Int. J. Therm. Sci..

[14] Hu H.W., Wang Q., Chen X.N., Li Q., Du M., Niu D. (2024). Droplet Condensation and Transport Properties on Multiple Composite Surface: A Molecular Dynamics Study. Front. Heat Mass Transf..

[15] Bai P., Zhou L., Du X. (2020). Effects of Surface Temperature and Wettability on Explosive Boiling of Liquid Argon Film on Copper Surface: A Molecular Dynamics Simulation Study. Int. J. Heat Mass Transf..

[16] Kim J. (2009). Review of Nucleate Pool Boiling Bubble Heat Transfer Mechanisms. Int. J. Multiph. Flow.

[17] Liang G., Mudawar I. (2018). Pool Boiling Critical Heat Flux (CHF)-Part 1: Review of Mechanisms, Models, and Correlations. Int. J. Heat Mass Transf..

[18] Kamatchi R., Venkatachalapathy S. (2015). Parametric Study of Pool Boiling Heat Transfer with Nanofluids for the Enhancement of Critical Heat Flux: A Review. Int. J. Therm. Sci..

[19] Lin L., Kedzierski M. (2019). Review of Low-GWP Refrigerant Pool Boiling Heat Transfer on Enhanced Surfaces. Int. J. Heat Mass Transf..

[20] Xu N., Liu Z., Yu X., Gao J., Chu H. (2024). Processes, Models and the Influencing Factors for Enhanced Boiling Heat Transfer in Porous Structures. Renew. Sustain. Energy Rev..

[21] Ghazivini M., Hafez M., Ratanpara A., Kim M. (2022). A Review on Correlations of Bubble Growth Mechanisms and Bubble Dynamics Parameters in Nucleate Boiling. J. Therm. Anal. Calorim..

[22] Inbaoli A., Sujith Kumar C., Jayaraj S. (2022). A Review on Techniques to Alter the Bubble Dynamics in Pool Boiling. Appl. Therm. Eng..

[23] Chen L., Kang Q., Mu Y., He Y.L., Tao W.Q. (2014). A Critical Review of the Pseudopotential Multiphase Lattice Boltzmann Model: Methods and Applications. Int. J. Heat Mass Transf..

[24] Tao W.Q., Chen L., Ling K., Chen Y.J. (2022). Some Advances in Numerical Simulation of Multiscale Heat Transfer Problems and Particularly for Boiling Heat Transfer. Annu. Rev. Heat Transf..

[25] Chen Y., Yu B., Lu W., Wang B., Sun D., Jiao K., Zhang W., Tao W. (2024). Review on Numerical Simulation of Boiling Heat Transfer from Atomistic to Mesoscopic and Macroscopic Scales. Int. J. Heat Mass Transf..

[26] Shavik S.M., Hasan M.N., Morshed A.K.M., Islam M.Q. (2015). Molecular Dynamics Study of Effect of Different Wetting Conditions on Evaporation and Rapid Boiling of Ultra-Thin Argon Layer over Platinum Surface. Procedia Eng..

[27] Cao Q., Shao W., Ren X., Ma X., Shao K., Cui Z., Liu Y. (2020). Molecular Dynamics Simulations of the Liquid Film Evaporation Heat Transfer on Different Wettability Hybrid Surfaces at the Nanoscale. J. Mol. Liq..

[28] Zhang L., Xu J., Liu G., Lei J. (2020). Nucleate Boiling on Nanostructured Surfaces Using Molecular Dynamics Simulations. Int. J. Therm. Sci..

[29] Zhou W., Han D., Ma H., Hu Y., Xia G. (2022). Molecular Dynamics Study on Enhanced Nucleate Boiling Heat Transfer on Nanostructured Surfaces with Rectangular Cavities. Int. J. Heat Mass Transf..

[30] Hu H.W., Lu Y., Guo L., Chen X., Wang Q., Wang J., Li Q. (2024). Effects of System Pressure on Nucleate Boiling: Insights from Molecular Dynamics. J. Mol. Liq..

[31] Li Y., Hu H.W. (2017). Effect of Wettability on Transport Performance of Nanoporous Ceramic Membranes. CIESC J..

[32] Hu H.W., Li Q., Liu S., Fang T. (2019). Molecular Dynamics Study on Water Vapor Condensation and Infiltration Characteristics in Nanopores with Tunable Wettability. Appl. Surf. Sci..

[33] Deng M.L., Li X.J., Ming P.J. (2026). Explosive Boiling of Liquid Hydrogen Molecular Dynamics Simulation: Microscopic Mechanisms and Effect of Surface Wettability. Int. Commun. Heat Mass Transf..

[34] Wang Y.H., Wang S.Y., Lu G., Wang X.D. (2018). Explosive Boiling of Nano-Liquid Argon Films on High Temperature Platinum Walls: Effects of Surface Wettability and Film Thickness. Int. J. Therm. Sci..

[35] Wang Y.H., Wang S.Y., Lu G., Wang X.D. (2019). Effects of Wettability on Explosive Boiling of Nanoscale Liquid Films: Whether the Classical Nucleation Theory Fails or Not. Int. J. Heat Mass Transf..

[36] Ilic M., Stevanovic V.D., Milivojevic S., Petrovic M.M. (2021). New Insights into Physics of Explosive Water Boiling Derived from Molecular Dynamics Simulations. Int. J. Heat Mass Transf..

[37] Mao Y., Zhang Y. (2014). Molecular Dynamics Simulation on Rapid Boiling of Water on a Hot Copper Plate. Appl. Therm. Eng..

[38] Fallahzadeh R., Bozzoli F., Cattani L., Azam M.W. (2024). Effect of Cross Nanowall Surface on the Onset Time of Explosive Boiling: A Molecular Dynamics Study. Energies.

[39] Morshed A.K.M.M., Paul T.C., Khan J.A. (2011). Effect of Nanostructures on Evaporation and Explosive Boiling of Thin Liquid Films: A Molecular Dynamics Study. Appl. Phys. A.

[40] Pillai R., Borg M.K., Reese J.M. (2018). Acoustothermal Atomization of Water Nanofilms. Phys. Rev. Lett..

[41] Liu H., Ahmad S., Chen J., Zhao J. (2020). Molecular Dynamics Study of the Nanoscale Boiling Heat Transfer Process on Nanostructured Surfaces. Int. Commun. Heat Mass Transf..

[42] Thompson A.P., Aktulga H.M., Berger R., Bolintineanu D.S., Brown W.M., Crozier P.S., in ‘t Veld P.J., Kohlmeyer A., Moore S.G., Nguyen T.D. (2022). LAMMPS—A Flexible Simulation Tool for Particle-Based Materials Modeling at the Atomic, Meso, and Continuum Scales. Comput. Phys. Commun..

[43] Stukowski A. (2010). Visualization and Analysis of Atomistic Simulation Data with OVITO-The Open Visualization Tool. Model. Simul. Mater. Sci. Eng..

[44] Abascal J.L.F., Vega C. (2005). A General Purpose Model for the Condensed Phases of Water: TIP4P/2005. J. Chem. Phys..

[45] Werder T., Walther J.H., Jaffe R.L., Halicioglu T., Koumoutsakos P. (2003). On the Water–Carbon Interaction for Use in Molecular Dynamics Simulations of Graphite and Carbon Nanotubes. J. Phys. Chem. B.

[46] He Y., Wang S., Tang Y.Z., Wu Z., Li W. (2023). Molecular Dynamics Simulation on Liquid Nanofilm Boiling over Vibrating Surface. Int. J. Heat Mass Transf..

[47] Gao D.Y., Han J.Y., Liu Z.W., He W., Sun Z., Zhao C., Bo H. (2025). Studies on Coupled Effects of Roughness and Wettability on Boiling Heat Transfer and Nucleation via Molecular Dynamics Simulation. Case Stud. Therm. Eng..

