# Supplementary material for: Coupled Effects of Wall Vibration and Surface Wettability on Nanoscale Liquid Film Boiling: A Molecular Dynamics Study
Source: Nanomaterials (Basel). 2026 Jul 31;16(15):944. doi: 10.3390/nano16150944 (PMC13468630; doi:10.3390/nano16150944)
Supplement: Supplementary file 1 [file nanomaterials-16-00944-s001.zip › nanomaterials-4386303-supplementary.pdf]

Supplementary Materials

Coupled Effects of Wall Vibration and Surface Wettability on Nanoscale Liquid-Film Boiling: A Molecular Dynamics Study

Haowei Hu, Zhenxin Chen, Feilong Zhao, Qin Li *, Lin Guo

S1. Contact-Angle Calibration Simulations

Additional equilibrium droplet simulations were performed to calibrate the apparent contact angle corresponding to the solid-liquid interaction parameter β. Three interaction strengths were examined, β = 0.013, 0.02, and 0.1. In this calibration, β denotes the O-Pt Lennard-Jones interaction energy parameter εO-Pt in eV, while the O-Pt size parameter was kept constant. The simulations were used only to establish the correspondence between β and surface wettability before the liquid-film boiling simulations.

The water droplet was initially placed above a Pt(100) substrate and equilibrated at 300 K. The apparent contact angle was then extracted from the axisymmetric r-z oxygen-density field. The oxygen number-density contour corresponding to half of the bulk oxygen density was used to identify the liquid-vapor interface, and the near-wall adsorbed layer was excluded from the fitting region. A circular fit of the interface was used to estimate the apparent contact angle relative to the Pt reference plane.

S1.1. Simulation Model

Table S1. System composition and initial droplet configuration used for contact-angle calibration.

Parameter	Value
Simulation box	200 × 200 × 200 Å^3
Total number of atoms	80,994
Number of water molecules	16,594
Number of water atoms	49,782
Number of Pt atoms	31,212
Initial droplet radius	50 Å
Initial droplet center	(100, 100, 62.8) Å
Highest Pt surface	z = 9.8 Å
Initial droplet-Pt gap	approximately 3 Å
Water molecular number density	0.031692 molecule/Å^3
Initial mass density	approximately 0.948 g/cm^3
Initial droplet volume	523,598.78 Å^3

Table S2. Pt substrate and water-model parameters.

Category	Parameter	Value
Pt substrate	Crystal structure	FCC
Pt substrate	Lattice constant	3.92 Å
Pt substrate	Surface orientation	Pt(100)
Pt substrate	Number of Pt layers	6
Pt substrate	Layer spacing	1.96 Å
Pt substrate	Pt atoms per layer	5,202
Pt substrate	Constrained layers	bottom 2 layers

Pt substrate	Mobile layers	upper 4 layers
Pt substrate	Pt atomic mass	195.084 amu
Pt substrate	Pt potential	Pt_u3.eam
Water model	Model	implicit four-site TIP4P/2005
Water model	O mass	15.9994 amu
Water model	H mass	1.00794 amu
Water model	O charge	-1.1128 e
Water model	H charge	+0.5564 e
Water model	O-H bond length	0.9572 Å
Water model	H-O-H angle	104.52°
Water model	O-M distance	0.1546 Å
Water model	O-O LJ ε	0.00802 eV
Water model	O-O LJ σ	3.1589 Å

S1.2. Solid-Liquid Interaction Parameters

Table S3. O-Pt interaction parameters and calibrated apparent contact angles.

β	εO-Pt (eV)	εO-Pt (kJ/mol)	σO-Pt (Å)	Apparent contact angle
0.013	0.013	1.254	2.815	130°
0.02	0.020	1.930	2.815	68°
0.1	0.100	9.649	2.815	approximately 0°

Only the O-Pt interaction energy parameter was varied among the three calibration cases. The H-Pt Lennard-Jones interaction was set to zero, and the O-Pt size parameter was fixed at 2.815 Å. Long-range electrostatic interactions were evaluated using PPPM/TIP4P with slab correction for the non-periodic direction.

S1.3. Contact-Angle Analysis

Table S4. Parameters used for density-field construction and contact-angle extraction.

Parameter	Setting
Analyzed atoms	oxygen atoms in water
Coordinate representation	axisymmetric r-z density field
Radial bin size	2 Å
z-direction bin size	2 Å
Smoothing method	Gaussian smoothing with σ = 1 bin
Bulk oxygen number density	0.0317 Å^{-3}
Interface threshold	0.01585 Å^{-3}
Pt reference plane	mean height of the uppermost Pt atomic layer
Near-wall excluded region	first 5 Å above the Pt reference plane
Interface fitting method	circular fitting
Contact-angle expression	θ = arccos[(zPt - zc)/R]

The resulting density fields show a clear transition from a lyophobic surface at β = 0.013 to a lyophilic surface at β = 0.02 and a nearly completely wetting surface at β = 0.1. These calibrated wettability states were used as the three representative solid-liquid interaction conditions in the main boiling simulations.

Supplementary Figures

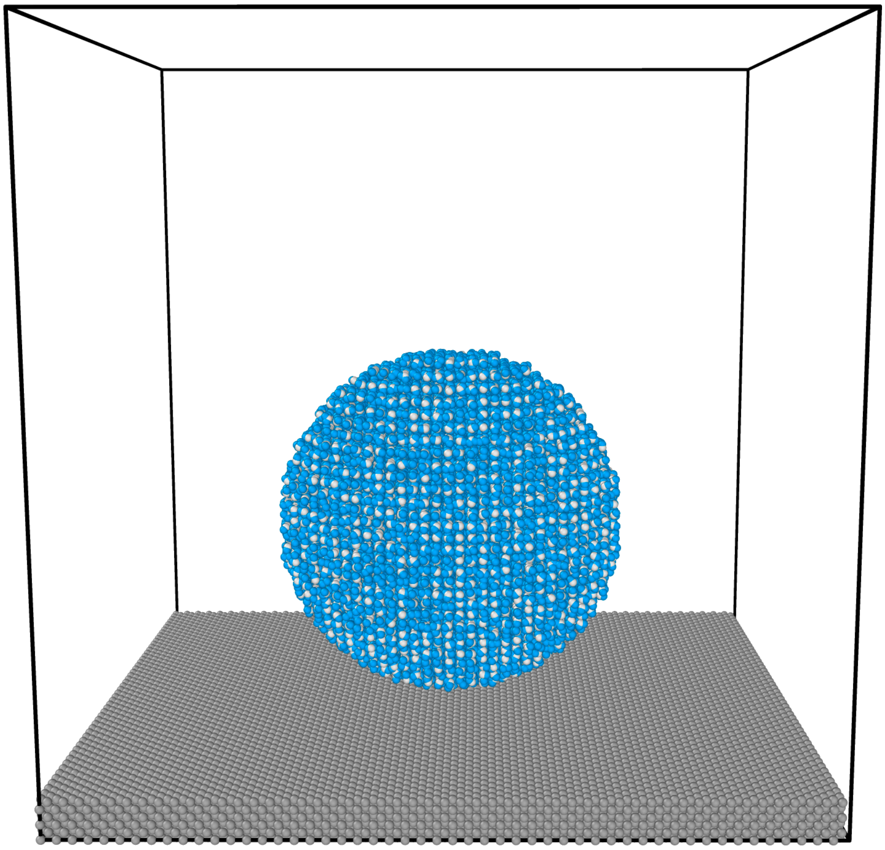

Figure S1. Initial droplet-on-Pt configuration used for contact-angle calibration.

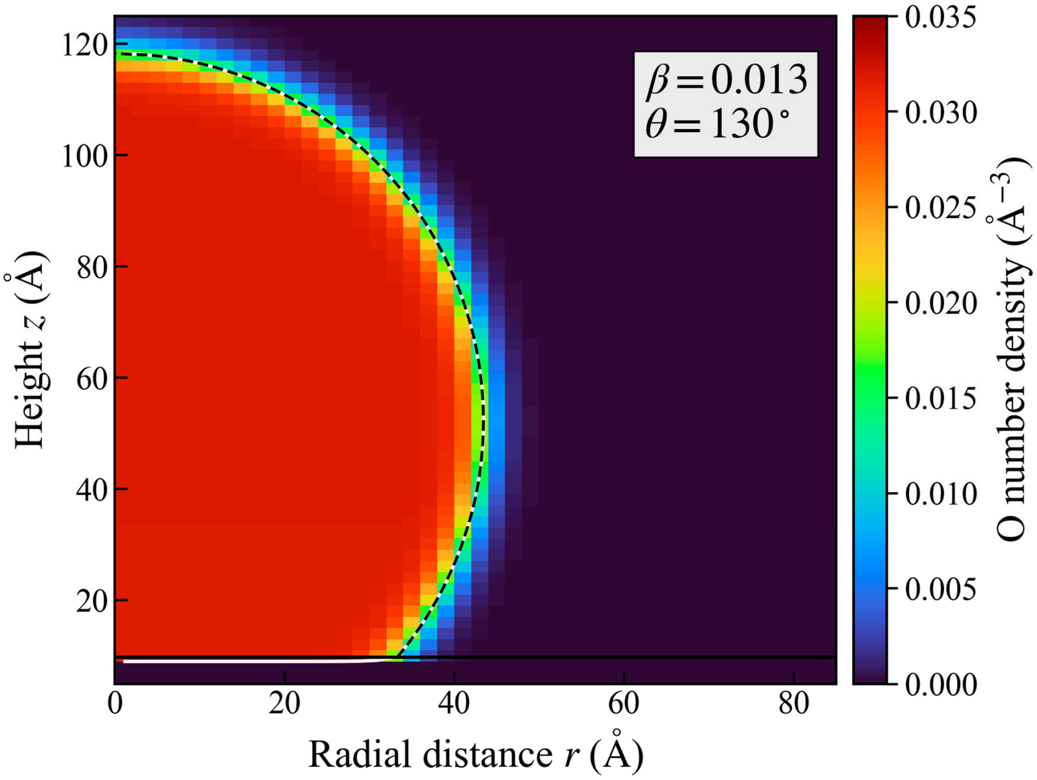

Figure S2. Axisymmetric oxygen-density cloud map and circular interface fitting for β = 0.013, corresponding to an apparent contact angle of 130°.

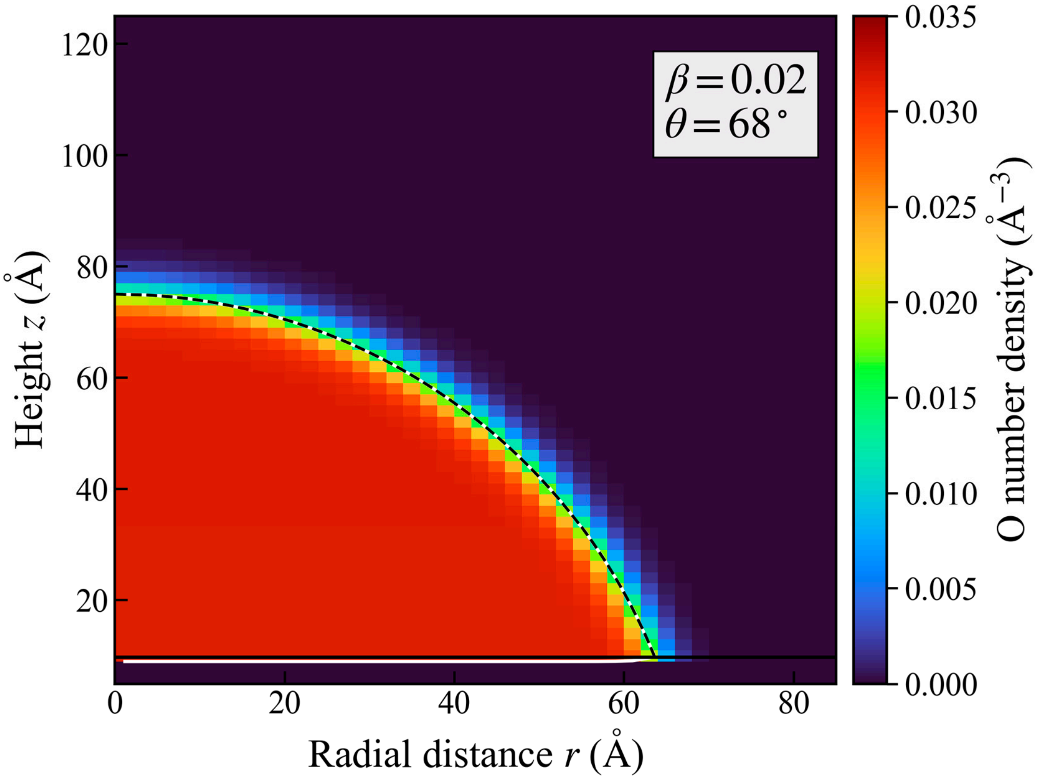

Figure S3. Axisymmetric oxygen-density cloud map and circular interface fitting for β = 0.02, corresponding to an apparent contact angle of 68°.

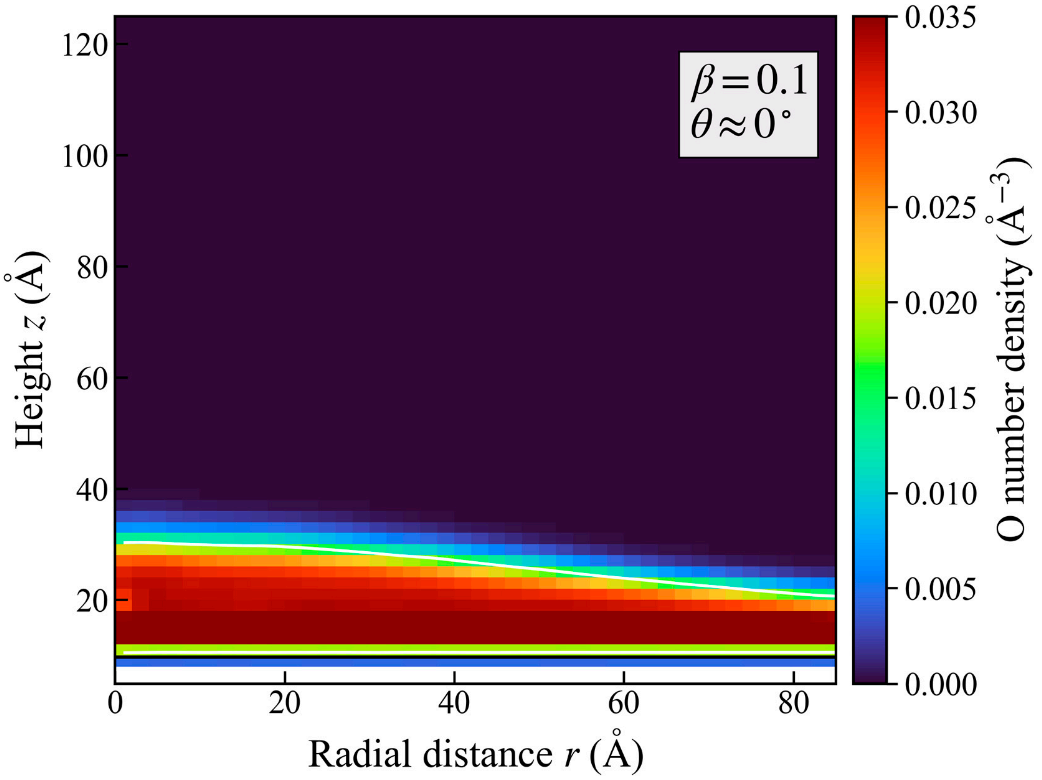

Figure S4. Axisymmetric oxygen-density cloud map for β = 0.1, corresponding to a nearly completely wetting surface with an apparent contact angle of approximately 0°.
